# Dermatoporosis in Upper Limbs Treated With Polymethylmethacrylate Microspheres Using the BioSculpt® Technique

**DOI:** 10.7759/cureus.43789

**Published:** 2023-08-20

**Authors:** Fernanda Bortolozo, Mariana Rinaldi, Priscila Souza, Ângela Schütz Paschoal, Gottfried Lemperle

**Affiliations:** 1 Dermatology, Fernanda Bortolozo Clinic, Balneário Camboriú, BRA; 2 Radiology, Global Health Center Clinic, São Paulo, BRA; 3 Biomedical Sciences, Fernanda Bortolozo Clinic, Balneário Camboriú, BRA; 4 Medicine, Fernanda Bortolozo Clinic, Balneário Camboriú, BRA; 5 Plastic Surgery, University of California San Diego, San Diego, USA

**Keywords:** skin disease/dermatology, age management medicine, geriatric dermatology, polymethylmethacrylate (pmma), ultrasound, dermatoporosis, bateman's purpura, actinic purpura, senile purpura, microspheres

## Abstract

Dermatoporosis is a syndrome of fragility or chronic cutaneous insufficiency. It presents with localized violaceous spots on the extensor face of the upper limbs of older people, with signs such as senile purpura, actinic purpura, or Bateman purpura, in addition to atrophy of the skin and subcutaneous tissue. These lesions can be painful and a source of morbidity. We report a case of an 80-year-old patient presented for the treatment of dermatoporosis in the upper limbs with polymethylmethacrylate (PMMA) using the BioSculpt®technique. The photographic and ultrasonographic clinical responses of the soft tissue were evaluated before and after nine months of treatment.

## Introduction

Dermatoporosis is a syndrome of chronic skin insufficiency that affects elderly individuals of both sexes. It is classified as primary or secondary due to the use of medications such as topical corticosteroids.

The primary presentation is attributed to skin aging and chronic photoexposure. Cutaneous atrophy, scars, and localized violaceous macules are present in the extensor areas of the hands and forearms. Minor traumas result in bruises, hematomas, abrasions, lacerations, ulcers, and blisters, which can progress to morbidity. The vascular fragility sign is termed senile purpura, Bateman's purpura, or actinic purpura [1].

The syndrome manifests through the loss of skin barrier functions and subcutaneous lipoatrophy. Blood infiltration due to vascular fragility, insufficient cushioning, inadequate inflammatory response, and dermoepidermal atrophy delays the absorption of extravasated blood, leading to hematoma formation and increased infection risk within subcutaneous collections [2].

The 2007 classification system [3] presents four stages depicting disease severity. Stage I evaluates skin thinning, senile purpura, and pseudoscars, categorized as IA, IB, IC, or ID based on lesion presence: IA: <10 pseudoscars and <10 senile purpura lesions; IB: <10 pseudoscars and >10 senile purpura lesions; IC: >10 pseudoscars and <10 senile purpura lesions; ID: >10 pseudoscars and >10 senile purpura lesions. Stage II has <10 lacerations, stage III has >10 lacerations, and stage IV includes any dissecting hematoma. Stage II indicates fewer than 10 lacerations, while stage III is marked by more than 10 lacerations. Stage IV is characterized by any dissecting hematoma.

The use of moisturizers and sunscreens is recommended for treating dermatoporosis. Mechanical protection such as clothing is also a consensus [4]. Topical retinol, for example, increases skin thickness, cell turnover, and hydration, although its effectiveness is hindered by initial burning and itching [3]. Prolonged use of topical corticosteroids exacerbates cutaneous atrophy [5].

A prior case report [6] details the use of calcium hydroxyapatite in forearm collagen biostimulation administered in two sessions for dermatoporosis treatment, with clinical improvement lasting up to six months.

Subcutaneous application of polymethylmethacrylate (PMMA) using the BioSculpt® technique [7] stimulates the production of new collagen around the microspheres, leading to long-lasting results. This method reduces the concentration of particles in the esthetic product from 30% to 10% and the viscosity of the carboxymethylcellulose hydrogel by including an anesthetic solution. As a result, PMMA implants can be used with a lower risk of nodule formation due to reduced product accumulation.

PMMA is a biocompatible synthetic polymer employed in subcutaneous, muscles, and bones in humans for more than 80 years [8,9]. The 40-micra microspheres are non-absorbable, providing a sustained stimulus for juxta-dermal neocollagenesis, enhanced nutritional support, and hydration through neoangiogenesis [10]. It improves subcutaneous tissue support by inducing new interlobular septa along the cannula trajectory. These properties may synergistically contribute to dermatoporosis treatment.

To expand treatment possibilities for primary and secondary dermatoporosis, we report a case of a patient undergoing collagen biostimulation treatment with PMMA using the BioSculpt® technique in the distal arms and forearms, with initial photographic evaluation, 45-day follow-up, and nine-month follow-up concluding with ultrasound assessment.

## Case presentation

An 80-year-old female presented with ecchymoses, hematomas, abrasions, blisters, and bleeding on her arms and forearms following minor trauma. She had used a topical retinoid for 30 days but discontinued it due to a burning sensation. Over the subsequent six months, her treatment regimen consisted of betamethasone cream on the left limb and daily application of moisturizer and sunscreen on both limbs. Protective measures, including wearing long-sleeved clothing, were adopted to mitigate potential trauma. Her medical history included thrombocytopenia and hypertension. Medications administered included enalapril, calcium, and vitamin D. Pre-procedure laboratory tests indicated normal results, except for mild thrombocytopenia.

**Figure 1:**
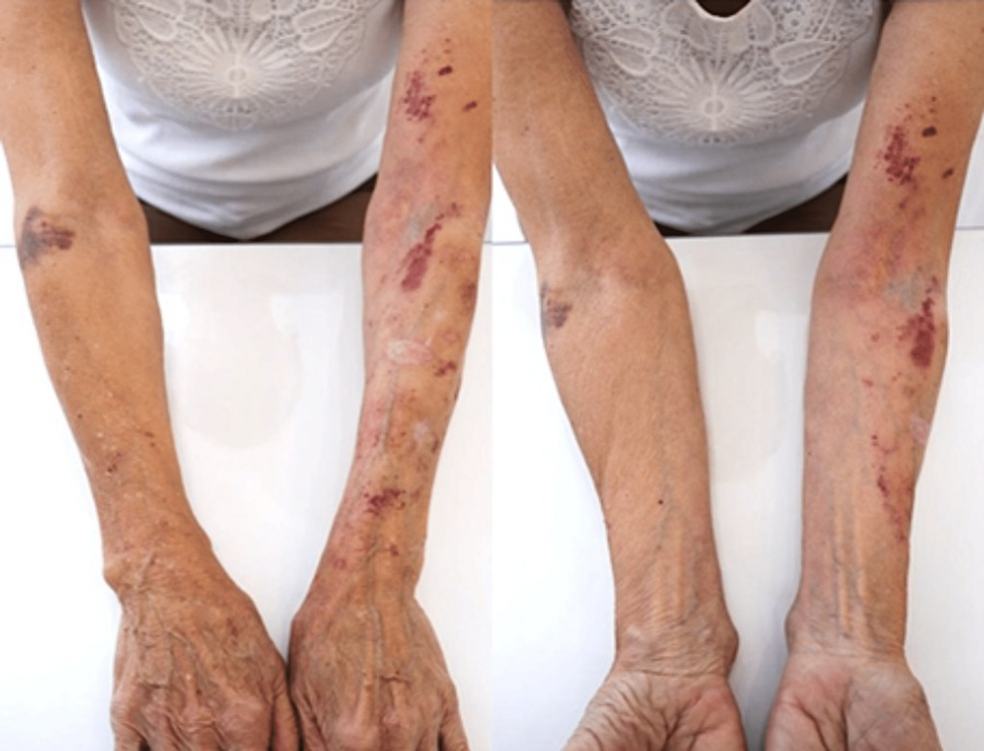
Upper limbs before treatment.

Examination revealed atrophic, hairless, translucent skin with circular and stellate depressed white scars, solar melanosis, guttate leukoderma, lipoatrophy, evident venous tracts, and purpuric, brown, and yellowish spots. The left side exhibited more pronounced lipoatrophy and a greater number of lesions (Figure 1).

**Figure 2:**
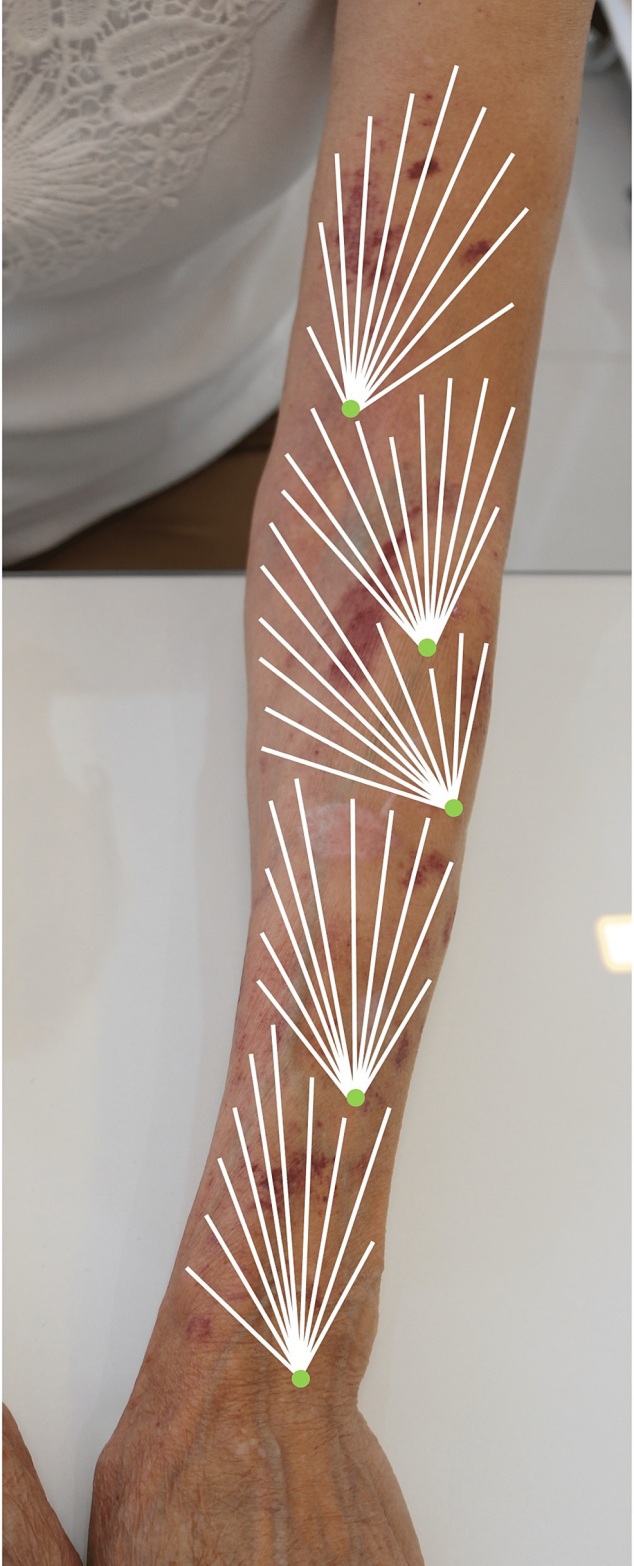
Fan retroinjection technique.

Sterile materials were used to execute the procedure and antisepsis protocols were observed. Each of the 10 entry points (Figure 2) received anesthesia with 0.1 mL of 2% lidocaine. A mixture of 15 mL of PMMA 30% Biossimetric® (MTC Medical Implants, Anápolis, Brazil) and 30 mL of anesthetic solution was prepared, resulting in a total volume of 45 mL of a 10% PMMA microspheres suspension. The recommended anesthetic solution, as outlined in the BioSculpt® technique [7], includes 250 mL of 0.9% saline solution, 20 mL of 2% lidocaine, 1 mL of 1 mg/mL adrenaline, and 10 mL of 8.4% sodium bicarbonate. At each entry point, 10 lines of retroinjection using the fan technique were uniformly administered, with each individual line receiving 0.45 mL. The treatment encompassed both the dorsal and ventral areas of the forearms and the distal arms within the juxta-dermal plane.

Subsequently, entry ports were dressed, light massage was performed, and analgesics were recommended to manage potential pain. The immediate post-procedure evolves with edema and eventual ecchymosis in the cannula entry ports. The use of a topical corticosteroid was proscribed while moisturizer and sunscreen were maintained.

**Figure 3:**
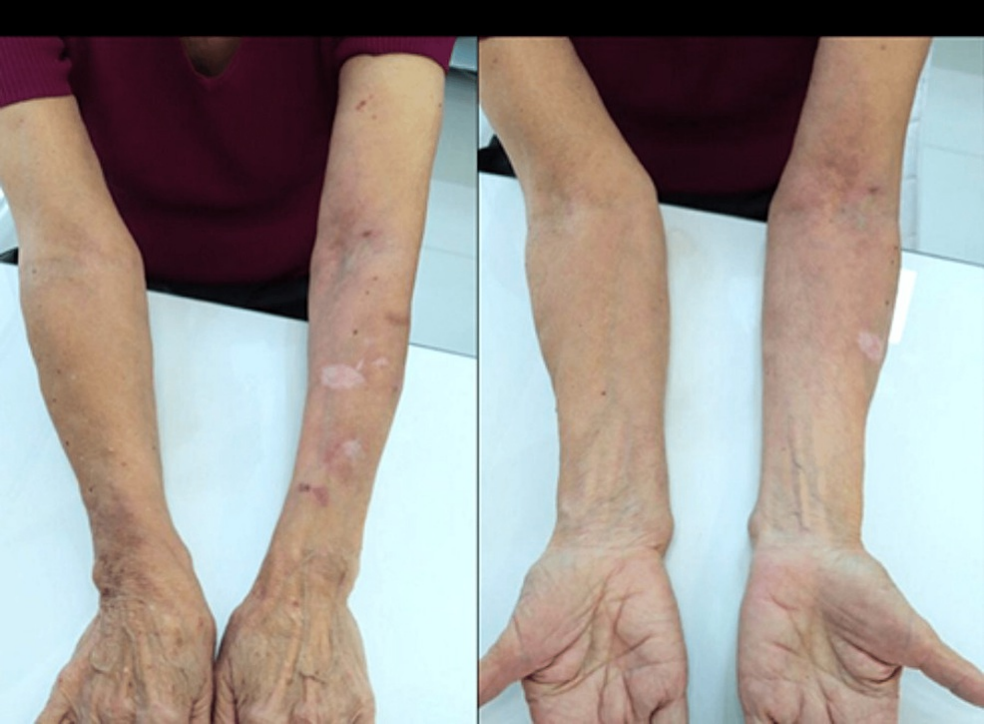
Forty-five days after treatment.

After 45 days of treatment, the patient exhibited two violaceous macules just on the left forearm dorsum and reduced skin translucency (Figure 3). Subsequently, disease classification regressed to stage 1A on the left side with fewer than 10 pseudoscars and senile purpura lesions, while the right side remained free of disease.

**Figure 4:**
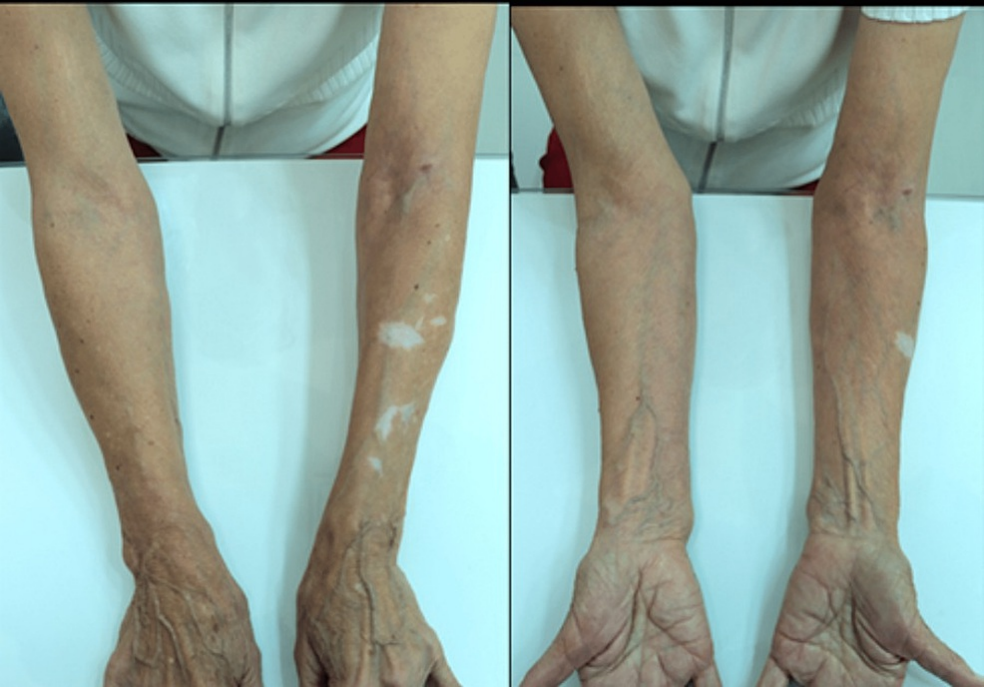
Nine months after treatment.

Nine months after the treatment, she returned with no new symptoms or lesions on the upper limbs (Figure 4).

**Figure 5:**
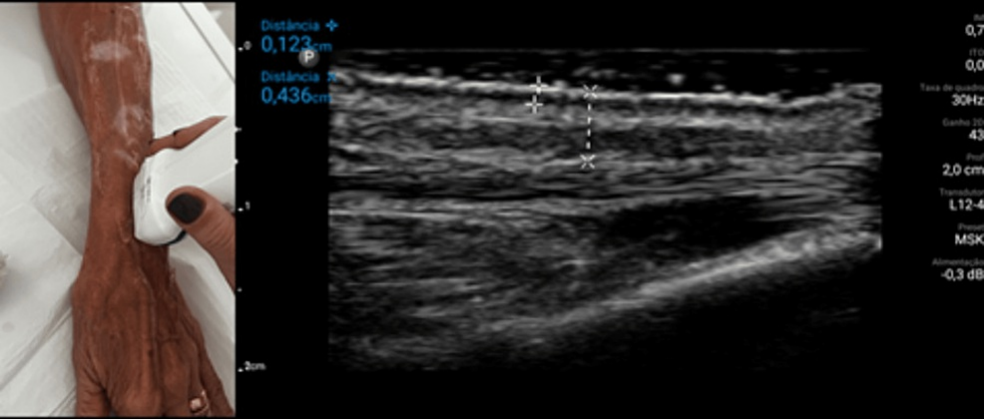
Ultrasound at nine months.

Physical examination revealed no visible or palpable nodules and ultrasonography (Lumify, Philips Medical Systems, Amsterdam, The Netherlands; linear 10 to 14 MHz) did not show any nodules either (Figure 5). There was no evidence of hyperechoic bright spots or posterior reverberation in the “comet tail” [11] characteristic of PMMA nodules on ultrasound.

The patient was discharged and was satisfied at nine months of treatment.

## Discussion

Dermatoporosis is a syndrome that affects the elderly and has no effective treatment so far [1,3-5]. The use of topical retinoids, moisturizers, and sunscreens acts only on the superficial layers of the skin [4], with no action on subcutaneous and dermal support or enhancement, or on vascular protection of the limbs [5]. The patient presented with bilateral primary dermatoporosis and secondary in the left upper limb with marked lipoatrophy (Figure 1). Despite noted improvement in a case using calcium hydroxyapatite [6], follow-up data post-particle resorption are lacking, impeding result persistence assessment.

The improvement in skin hydration, reduction of translucency, subcutaneous and dermal support, increased resistance to minimal traumas, and absence of new purpuric lesions were already evident at 45 days (Figure 3) and confirmed at nine months (Figure 4) of the treatment with PMMA microspheres in the BioSculpt® technique [7] (Figure 2) is probably due to the inherent and persistent reactions already established in the literature about PMMA implantation. In the presented case, an additional benefit of the treatment was bleeding prevention in a patient with thrombocytopenia. Although the high-frequency ultrasound [11] probe has a greater indication in skin studies, it was useful in confirming the absence of nodules in the subcutaneous tissue (Figure 5) of the patient with dermatoporosis.

## Conclusions

PMMA in the BioSculpt® technique emerges as a promising treatment option for upper limb dermatoporosis. Complete regression of disease signs and symptoms after nine months of treatment could represent a novel approach to disease management. Photographic evidence was presented, and nodules were not visualized in the ultrasound images. New studies with a larger number of patients, the use of high-frequency ultrasound, and longer follow-up will elucidate the effectiveness and safety of the treatment.
